# Magnesium Sulfate-Induced Myasthenic Crisis in Pregnancy: A Case Report

**DOI:** 10.7759/cureus.110900

**Published:** 2026-06-15

**Authors:** Lauren A Gould, Andrea Lorenz, Selena Nicotra, Vashun Rodriguez

**Affiliations:** 1 Emergency Medicine, Lakeland Regional Health Medical Center, Lakeland, USA

**Keywords:** intravenous immunoglobulin (ivig), magnesium sulphate, myasthenia gravis (mg), myasthenic crisis, preeclampsia (pe), seizure prevention

## Abstract

Myasthenia gravis (MG) is an autoimmune disorder characterized by antibodies targeting acetylcholine receptors (AChR) or muscle-specific kinase (MuSK) at the neuromuscular junction, resulting in fluctuating skeletal muscle weakness. Preeclampsia is an obstetric complication defined as new-onset hypertension and proteinuria, or new-onset hypertension with evidence of end-organ dysfunction with or without proteinuria, typically presenting after 20 weeks gestation or within six weeks postpartum.

We report a 37-year-old woman at 19 weeks' gestation who developed a myasthenic crisis following administration of intravenous magnesium sulfate for suspected preeclampsia.

When there is concern for preeclampsia in pregnant patients with MG, alternative treatments to magnesium sulfate should be utilized to avoid exacerbating or triggering a myasthenic crisis. In pregnant patients with MG, alternatives to magnesium sulfate should be considered for seizure prophylaxis and management because magnesium may precipitate or worsen myasthenic crisis. Hydralazine or nifedipine are considered first-line antihypertensive therapies in pregnant patients with MG; however, labetalol can also be used with caution because it may exacerbate MG symptoms.

## Introduction

Myasthenia gravis (MG) is a chronic autoimmune disorder caused by antibodies to muscle nicotinic acetylcholine receptors (nAChR) or muscle-specific kinase (MuSK) targeting the postsynaptic membrane at the neuromuscular junction that impairs neuromuscular transmission and causes fluctuating muscle weakness [1,2]. It commonly affects voluntary muscles, causing bulbar symptoms such as ptosis, diplopia, and dysphagia. Symptoms typically improve with rest and worsen with continued use, causing worsening muscle fatigue. One of the most severe and life-threatening complications of MG is acute respiratory failure due to respiratory muscle weakness, requiring endotracheal intubation and invasive mechanical ventilation. Standard treatment includes acetylcholinesterase inhibitors (i.e., pyridostigmine or neostigmine), corticosteroids (i.e., prednisone), steroid-sparing immunosuppressants (i.e., azathioprine, cyclosporine, mycophenolate mofetil, methotrexate, and tacrolimus), and thymectomy [1,3]. For refractory MG or myasthenic crisis, treatment options include rituximab, intravenous immunoglobulin (IVIG), or plasma exchange (PLEX) [1,3].

Preeclampsia is characterized by new-onset hypertension after 20 weeks' gestation or within 6 weeks postpartum, accompanied by proteinuria or evidence of maternal end-organ dysfunction [4,5]. Eclampsia is preeclampsia accompanied by seizure activity. Hypertension in pregnancy is defined as a systolic blood pressure >140 mmHg or a diastolic blood pressure >90 mmHg. End-organ dysfunction includes thrombocytopenia (platelet count <100,000/µL), renal insufficiency (serum creatinine >1.1 mg/dL or doubling of the serum creatinine concentration), elevated liver enzymes to twice the normal level, pulmonary edema, visual disturbances, or new-onset headaches refractory to medication [5]. Management of preeclampsia includes antihypertensives that are safe in pregnancy (i.e., labetalol, hydralazine, and nifedipine), magnesium sulfate for seizure prophylaxis, and diuretics (i.e., furosemide) [4,5]. Magnesium impairs neuromuscular transmission and is generally contraindicated in patients with MG because it can worsen weakness and precipitate a myasthenic crisis. Beta-blockers such as labetalol can also worsen MG symptoms and should be used with caution [3].

We present the case of a 37-year-old woman at 19 weeks' gestation who presented with a myasthenic crisis after receiving IV magnesium sulfate for possible preeclampsia.

## Case presentation

A 37-year-old G5P1 woman at 19 weeks' gestation with known MG, prescribed mycophenolate, prednisone, and pyridostigmine, presented as a transfer from another hospital for hypertension and progressive generalized weakness. Outside-hospital obstetric ultrasonography demonstrated a viable intrauterine pregnancy consistent with 19 weeks' gestation. Laboratory evaluation demonstrated normal renal function, liver enzyme levels, platelet count, and coagulation studies, as shown in Table 1. Urinalysis was consistent with a urinary tract infection (UTI), and the patient received 1 g of IV ceftriaxone at the referring hospital for an acute UTI. Due to hypertension (documented systolic blood pressure >160 mmHg and diastolic blood pressure >100 mmHg) and proteinuria, she also received 4 g of IV magnesium sulfate for presumed preeclampsia before transfer.

**Table 1:** Patient laboratory results. Summary of the patient's initial laboratory results obtained in the emergency department. BUN, blood urea nitrogen; GFR, glomerular filtration rate; WBC, white blood cell; RBC, red blood cell

Laboratory test	Result	Measurement
WBC count	9.31	10^3^/µL
RBC count	3.73	10^6^/µL
Hemoglobin	10.2	g/dL
Hematocrit	32.1	%
Mean corpuscular volume	86.1	fL
Platelets	340,000	/µL
Glucose	94	mg/dL
Sodium	136	mmol/dL
Potassium	3.9	mmol/dL
Chloride	99	mmol/dL
Bicarbonate	24.0	mmol/dL
Creatinine	0.57	mg/dL
BUN	9	mg/dL
GFR	>60	mL/ minute
Anion gap	13	-
Total protein	6.3	g/dL
Albumin level	3.48	g/dL
Bilirubin total	<0.2	mg/dL
Bilirubin direct	<0.1	mg/dL
Alk Phos	66	U/L
AST	16	U/L
ALT	8	U/L
Lipase	26	U/L
Magnesium	2.6	mg/dL
TSH	3.21	µlU/mL
Free T3	3.98	pg/mL
Free T4	1.00	ng/dL
Cortisol	21.8	µg/dL
Urinalysis	Turbid, pale yellow. Glucose: negative. Bilirubin: negative. Ketones: negative. Specific gravity: 1.013. Blood: 2+. pH: 6. Protein: 2+. Nitrite: positive. Leukocyte esterase: 3+. Many RBCs. Too numerous to count WBCs. Many bacteria. Few epithelial cells. Few hyaline casts. Mucus: 1+	

Upon arrival at our hospital, the patient complained of a headache, and her initial blood pressure was 156/94 mmHg. She had bilateral ptosis on examination and complained of dysphagia, which she stated began immediately after receiving the magnesium sulfate infusion at the previous hospital. She initially sought evaluation at the transferring hospital for lower abdominal pain and concern for her unborn child's well-being. She denied vaginal bleeding, loss of fluid, chest pain, shortness of breath, and urinary symptoms.

The patient stated that she was not currently taking her prescribed medications due to a lack of insurance. Bedside Doppler fetal heart tones were obtained, with a fetal heart rate of 158 beats per minute. Negative inspiratory force (NIF) ranged from −15 to −25 cm H₂O, indicating significant respiratory muscle weakness despite preserved oxygenation and the absence of overt respiratory distress. Both neurology and obstetrics were consulted, and the neurologist suggested either IVIG, if deemed safe in pregnancy, or plasma exchange. Following multidisciplinary discussion among the neurology, obstetrics, and pharmacy teams, IVIG (0.4 g/kg/day) was initiated. Neurology recommended a five-day course of IVIG. Endotracheal intubation was deferred because the patient was maintaining airway protection, generated a strong cough, spoke in complete sentences, and showed no clinical signs of impending respiratory failure. She was admitted to the intensive care unit (ICU) for myasthenic crisis and close monitoring in case of respiratory decline.

Upon admission to the ICU, the patient was restarted on her previously prescribed home dose of prednisone 40 mg daily and pyridostigmine 60 mg three times daily. Due to fetal risks, including teratogenicity and spontaneous abortion, mycophenolate was not resumed. Outpatient records from a hospital frequently visited by the patient confirmed positive acetylcholine receptor (AChR) antibody testing. Further evaluation favored chronic hypertension rather than preeclampsia based on documented antecedent hypertension and the absence of laboratory evidence of severe features. The diagnosis of preeclampsia should not be made before 20 weeks' gestation. She was treated initially with IV labetalol and then transitioned to nifedipine 30 mg twice daily. Urine culture grew multidrug-resistant Escherichia coli, and the patient was transitioned to IV meropenem based on culture sensitivities.

The patient’s NIF improved to between -30 and -35 cm H₂O, and she was transferred from the ICU to the intermediate care unit. Daily NIF measurements remained stable throughout the remainder of her admission, ranging from -30 to -35 cm H₂O. The patient completed her course of IVIG and antibiotics and was discharged with outpatient follow-up with neurology and obstetrics.

## Discussion

Several factors likely contributed to the patient's decompensation, including active infection, interruption of maintenance MG therapy, and exposure to IV magnesium sulfate. Notably, the patient reported that dysphagia and weakness developed immediately following the magnesium infusion. Patients in myasthenic crisis are at risk of respiratory failure, and NIF and forced vital capacity (FVC) can be measured as markers of respiratory status and trended to monitor for respiratory decline. Serial NIF and FVC measurements are commonly used to monitor respiratory function in myasthenic crisis. In this case, only serial NIF measurements were consistently documented.

Several medications may exacerbate MG, including aminoglycosides, fluoroquinolones, macrolides, beta-blockers, magnesium-containing agents, and certain antiarrhythmics [1-3]. Careful medication review is therefore essential. Prior case reports have described worsening neuromuscular weakness and respiratory compromise following magnesium administration in patients with MG, supporting avoidance whenever suitable alternatives exist [2,6,7]. Magnesium sulfate should only be administered to patients with MG in life-threatening situations such as severe hypomagnesemia; however, it should be administered at a slower rate, and patients should be closely monitored for respiratory distress secondary to worsening muscle weakness.

Alternatives to magnesium sulfate administration in pregnant patients with MG and preeclampsia include treating seizures with benzodiazepines, providing seizure prophylaxis with barbiturates or phenytoin, and controlling blood pressure with hydralazine or nifedipine [1,3]. Labetalol may be used; however, beta-blockers can theoretically worsen MG-related muscle weakness, so nifedipine and hydralazine are preferred [8,9].

Most women with MG experience successful pregnancies, although rates of cesarean delivery are higher than in the general obstetric population (17% vs. 8.6%, respectively) [10,11]. Approximately 10% of infants born to mothers with MG experience transient muscle weakness due to antibodies against AChR or MuSK that cross the placenta from the maternal circulation to the fetus [10]. Symptoms may include generalized hypotonia, poor sucking due to reduced muscle strength, dysphagia, and a weak cry. These symptoms of neonatal myasthenia are typically transient, most pronounced during the first week of life, and resolve within the first four weeks of life [10].

## Conclusions

Magnesium sulfate may precipitate or exacerbate a myasthenic crisis in patients with underlying MG. This case highlights the importance of recognizing MG as a relative contraindication to magnesium administration and of considering alternative approaches when treating pregnant patients with suspected preeclampsia. Early multidisciplinary involvement and close respiratory monitoring are essential when clinical deterioration occurs.
